# *π*-Conjugated Polymers and Their Application in Organic and Hybrid Organic-Silicon Solar Cells

**DOI:** 10.3390/polym14040716

**Published:** 2022-02-13

**Authors:** Siyabonga B. Mdluli, Morongwa E. Ramoroka, Sodiq T. Yussuf, Kwena D. Modibane, Vivian S. John-Denk, Emmanuel I. Iwuoha

**Affiliations:** 1 Sensor Laboratories (SensorLab), University of the Western Cape, Robert Sobukwe Road, Bellville, Cape Town 7535, South Africa; 3693152@myuwc.ac.za (M.E.R.); 3773164@myuwc.ac.za (S.T.Y.); 3377067@myuwc.ac.za (V.S.J.-D.); 2 Department of Chemistry, School of Physical and Mineral Science, University of Limpopo, Sovenga, Polokwane 0727, South Africa; kwena.modibane@ul.ac.za

**Keywords:** heterojunction solar cell, hole transporting layer, n- and p-type doping, organic photovoltaic cell, *π*-conjugated polymer

## Abstract

The evolution and emergence of organic solar cells and hybrid organic-silicon heterojunction solar cells have been deemed as promising sustainable future technologies, owing to the use of *π*-conjugated polymers. In this regard, the scope of this review article presents a comprehensive summary of the applications of *π*-conjugated polymers as hole transporting layers (HTLs) or emitters in both organic solar cells and organic-silicon hybrid heterojunction solar cells. The different techniques used to synthesize these polymers are discussed in detail, including their electronic band structure and doping mechanisms. The general architecture and principle of operating heterojunction solar cells is addressed. In both discussed solar cell types, incorporation of *π*-conjugated polymers as HTLs have seen a dramatic increase in efficiencies attained by these devices, owing to the high transmittance in the visible to near-infrared region, reduced carrier recombination, high conductivity, and high hole mobilities possessed by the p-type polymeric materials. However, these cells suffer from long-term stability due to photo-oxidation and parasitic absorptions at the anode interface that results in total degradation of the polymeric p-type materials. Although great progress has been seen in the incorporation of conjugated polymers in the various solar cell types, there is still a long way to go for cells incorporating polymeric materials to realize commercialization and large-scale industrial production due to the shortcomings in the stability of the polymers. This review therefore discusses the progress in using polymeric materials as HTLs in organic solar cells and hybrid organic-silicon heterojunction solar cells with the intention to provide insight on the quest of producing highly efficient but less expensive solar cells.

## 1. Introduction

Over the past centuries, the demand for new technologies based on alternative renewable energy sources has seen an exponential growth due to the alarming rate of increasing global population, the current global energy catastrophe, the unceasing consumption of fossil fuel storages, and the emission of greenhouse gases to the atmosphere because of the combustion of fossil fuels. This growing demand for sustainable and clean energy resources has significantly led to increased attention into the scientific research, development, and manufacturing of various new technologies based on renewable energy resources [1]. Amongst the various existing sustainable energy sources, solar energy has gained the most recognition, due to the continuous availability of sunlight; economic viability, sustainability, and its promise to reduce greenhouse gases, make it an ideal approach for addressing problems associated with energy and the environment, through a photoelectric effect phenomenon utilizing photovoltaic devices [2,3,4]. Photovoltaic devices are commonly known as devices focused on converting solar irradiation energy directly into electrical energy by exploiting the photoelectric effect exhibited by metals and inorganic semiconductors [5]. Traditional inorganic silicon-based modules, also known as first generation solar cells, currently account for the broad majority of solar cell technology in both the residential and industrial markets. Recently, the power conversion efficiencies (PCE) competitive to the traditional solar cells have been realized by utilizing the carrier-selective layer’s approach on crystalline silicon wafers with high-quality Float Zone (FZ) [6,7]. The architecture of this solar cell comprises of an intrinsic hydrogenated amorphous silicon ((*i*) a-Si:H) layer placed either on both sides or one side of the FZ silicon wafer, acting as a passivation layer, prior to the formation of carrier-selective contact. This type of silicon-based technology has led to a record breaking PCE of 26.6%, formed from the blend of interdigitated back contact and heterojunction concepts [8], which is near to the silicon solar cell theoretical limit PCE of 29.1% [9]. Despite the significant performance progress and higher attained efficiencies by silicon-based solar cells, their large-scale industrial production and commercialization is restricted and hampered by the complexity in fabrication processes and the involved number of steps, which makes the solar panels extremely expensive. These drawbacks and limitations experienced by silicon PV cells and the inability to provide cost-effective energy has shifted research to focus on the development and exploration of alternative materials such as cadmium indium gallium diselenide (CIGS) and cadmium telluride (CdTe), widely known as second generation thin-film photovoltaics, to decrease the manufacturing cost and increase manufacturing and production. These thin-film photovoltaic devices have seen a production of higher efficiencies for single-junction terrestrial cells of 21.4% and 21.6% for CdTe and CIGS, respectively [10], which are very close to the produced efficiency by silicon solar cells. These cells are relatively cheaper and more stable than silicon based solar cells; however, their efficiencies require further improvements for large-scale industrial production in order to compete with silicon in the photovoltaic market. Alternatively, plastic solar cells, also known as third-generation solar cells, include perovskite solar cells (PSCs), organic solar cells (OSCs), and dye-sensitized solar cells (DSSCs), have gained increasing recognition owing to their flexibility, low-cost manufacturing or production, ease of synthesis of the materials, and the simplicity of their fabrication processes. Currently, the output of these devices has attained efficiencies for single-junction terrestrial cells of 13% [11], 16–18.1% [11,12,13,14,15], and 25.5% [11,16] for dye-sensitized solar cells, organic solar cells, and perovskite solar cells, respectively.

**Scheme 1:**
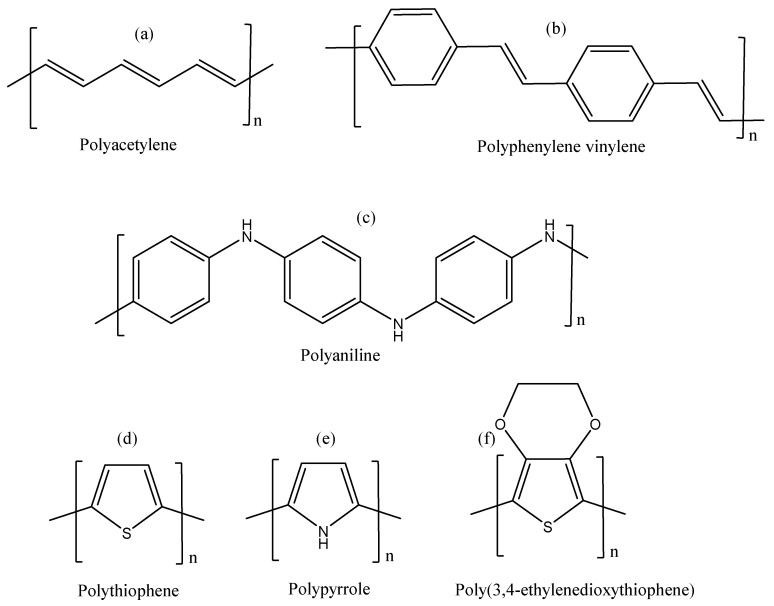
Chemical structures of the mostly studied and applied π-conjugated polymers. (**a**) Polyacetylene, (**b**) polyphenylene vinylene, (**c**) polyaniline, (**d**) polythiophene, (**e**) polypyrrole, and (**f**) poly(3,4-ethylenedioxythiophen).

Organic solar cells have been receiving increased scientific attention recently, due to the low production costs, reduced complexity of the fabrication processes, improved material processability and the use of π-conjugated polymers. Research has shifted focus towards the area of modeling, fabrication, design and comprehensive investigations of the applications and properties of π-conjugated polymers, since the breakthrough discovery of chemical doped polyacetylene possessing electrical conductivity [17,18]. These have gained widespread considerable recognition because of their superior advantages such as the ability to tailor their electrical and optical properties through structural modifications, ease of synthesis, flexibility, low production cost and ease of processability [19]. These first-class properties have therefore qualified conjugated polymers to be extensively explored in applications of optoelectronic devices such as organic light-emitting diodes (LEDs), electrochromic devices, photovoltaics, nonlinear optical devices, field-effect transistors (FETs), memory storage devices, chemo-and bio-sensors, and energy storage photodetectors [20,21,22,23,24]. The diverse technological application of conjugated polymers is owing to the addition of side-chains in these polymeric materials, which reduces backbone stiffness, enhances their thermal stability and solubility, and allows the construction of nanostructured layers using less expensive, easy solution-processable deposition technique such as spin-coating [25]. In addition, conjugated polymers (Scheme 1) demonstrating a series of interchanging satisfactory acceptor and donor units in the polymer main segments, to some extent, can exhibit low band-gap energy values, because of the intramolecular charge transfer interactions. Therefore, conjugated systems consisting of alternating single and double bonds are considered as advanced class of materials in the field of electronics and photonics owing to their rich π-excessive nature [26,27,28].

For the most part, conjugated polymers have found numerous utilities in different types of PV devices and architectures. These include, (1) wide use in production of organic solar cells as hole transporting layers (HTLs) [29] and donor materials [30], (2) use in electrochemical dye-sensitized solar cells as host electrolytes [31], (3) emitters in heterojunction silicon solar cells in place of highly doped p-type hydrogenated amorphous silicon [32] and (4) p-type HTLs in perovskite solar cells [33]. Herein, the various synthetic techniques of conjugated polymers are presented, including their electronic band structure and doping mechanisms, together with the application of conjugated polymers as HTLs in organic solar cells, and organic-silicon hybrid heterojunction solar cells are discussed in detail.

## 2. Electronic Structure and Doping Mechanisms in π-Conjugated Polymers

### 2.1. The Electronic Structure

So as to achieve a fair impression on the origin of electrical conductivity and superior optical properties such as low band gaps in π-conjugated polymeric materials, it is imperative to understand the hybridization and electronic configuration exhibited by conjugated polymers in comparison to non-conjugated polymers. As it is well known, the element carbon has the electronic structure, 1s^2^2s^2^2p^2^, with four valence electrons available to form four bonds with neighboring adjacent atoms. In non-conjugated polymers, carbon atoms in the polymer main chain are sp^3^ hybridized, and each carbon atom forms four covalent σ-bonds with adjacent neighbouring atoms. In such non-conjugated polymers, the electrons are strongly localized as each carbon atom utilizes all its four valance electrons, as the main chain of the polymer backbone consists of only σ bonds. Consequently, non-conjugated polymers are electrically insulating in nature as a result of low mobility caused by the unavailability of free electrons to move along the polymer backbones that are responsible for inducing electrical conductivity. Additionally, σ-bonded polymers, generally have larger electronic energy band gaps, *E_g_* (σ), due to the long single bonds, inhibit photon absorption in the elecctromagnetc spectrum from visible-to-near infrared region, thus becoming electronically insulating. For example, polyethylene, defined by -(CH_2_–CH_2_)_n_- monomer repeating units, has been well studied and found to be electrically insulating with an optical band gap of 8 eV [34]. 

In conjugated polymers, carbon atoms in the polymer main chain are sp^2^ hybridized and each carbon atom forms three covalent σ-bonds with adjacent neighbouring atoms, and one remaining unhybridized 2p_z_ atomic orbital (orthogonal to the three σ-bonds), which has an unpaired electron that overlaps with the unpaired electron of the neighbouring 2p_z_ atomic orbital of sp^2^ hybridized carbon atom. The continuous overlap of these 2p_z_ atomic orbitals lead to the formation of π-bonds/electrons delocalized on the polymer backbone. Therefore, due to this electronic delocalization, semiconducting characteristics are realized as a result of high mobility induced by the presence of free π-electrons that are moving along the polymer backbone, thus causing electrical conductivity. In addition, π-bonded polymers have relatively small band gaps compared to σ-bonded polymers, *E_g_* (π) < *E_g_* (σ), due to the presence of the short double bonds, leading to optical absorption from visible-to-near infrared lower photon energies, with low-energy electronic excitations, and semiconductor behavior [25]. For example, polyacetylene (see structure in Scheme 1), defined by –(CH=CH)_n_– monomer repeating units, has been well studied and demonstrated a semiconductor character with an optical band gap of 1.5 eV [35]. 

### 2.2. Doping Mechanisms of Conjugated Polymers

As it is well known, π-conjugated polymeric materials in their intrinsic condition are neutral and can be either insulators or semiconductors with very little conductivity due to low mobility rates on the polymer’s main chain [24]. However, the electrical properties, and hence the semiconductor characteristic improvements strongly depend on doping. Doping is a phenomenon where impurities or foreign materials are introduced in the intrinsic semiconductor, conjugated polymers in this context, for the sole intention of regulating its electrical, structural, and optical properties, thereby referred to as an extrinsic semiconductor. Upon doping, which results in chemical changes within the polymer matrix, charges are generated and propagate through the polymer chemical structure. Subsequently, the conductivity significantly elevates by numerous orders of magnitude due to the reduction between the lowest unoccupied molecular orbital (LUMO) and the highest occupied molecular orbital (HOMO) energy level band gap from 3–6 eV in neutral stable (Undoped) conjugated polymers to 1–4 eV in doped conjugated polymers [36,37]. For instance, the conductivity has seen a sharp increase from 10^−2^–10^−5^ S/cm in undoped conjugated polymers to 10^−5^–10^3^ S/cm in doped conjugated polymers, which is close to the metallic region [37,38]. Doping in conjugated polymers can be carried out in different routes using techniques such as electrochemical (redox) doping, chemical doping (gaseous and solution), in-situ doping, charge-injection, and radiation-induced doping [39,40]. It can be further distinctly classified according to the electron transfer type such as p-type doping using oxidizing agents and n-type doping using reducing agent, and according to the chemical nature of the dopant, such as organic, inorganic, and polymeric [41].

π-Conjugated polymer + dopant ⟶ electrically conducting polymer

**Scheme 2:**
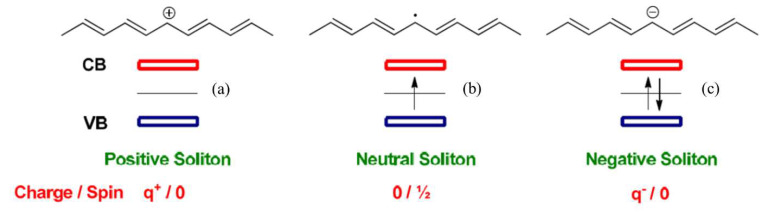
The domain boundary (neutral soliton) of two degenerate ground states of t-PA and charged solitons (positive and negative) [24]. (**a**) positive soliton, (**b**) neutral soliton, and (**c**) negative solution.

As a result of doping, the introduction of foreign materials, either acceptor or donor species, into the conjugated polymeric matrix results in the incorporation of defects. The created defects are labeled as bipolaron, polaron and soliton. The formation of neutral soliton, positive soliton and negative soliton of polyacetylene (PA) in its trans-configuration is depicted in Scheme 2. 

**Figure 1:**
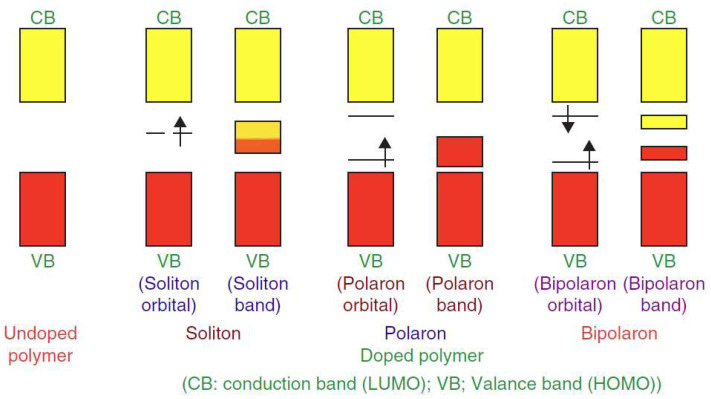
Doped and undoped (soliton, polaron, bipolaron) conjugated polymer orbital and band structures. Reproduced with permission from reference [43], copyright (2013), John Wiley and Sons.

A neutral soliton is formed by the donation of one single electron by the dopant to the polymer or the acceptance of one single electron from the polymer to the dopant, with the unhybridized 2p_z_ orbital having an unpaired electron and a half-occupied band gap with reference to the conduction band (CB) and valence band (VB). In the case of charged solitons, a positive soliton is formed when a pair of electrons is accepted by the dopant (p-type doping) which results in an empty mid-gap; a negative soliton is formed when a pair of electrons is donated by the dopant (n-type doping) resulting in a full mid-gap with two electrons. The soliton state exhibits a form of degenerate ground state owing to the isolation of the two-phased bonding electrons in the reverse direction; however, possessing the exact indistinguishable energy state. As a result, a new localized electronic state responsible for conducting electrical energy, called a soliton band, takes shape in the center of the band gap (see Figure 1).

**Scheme 3:**
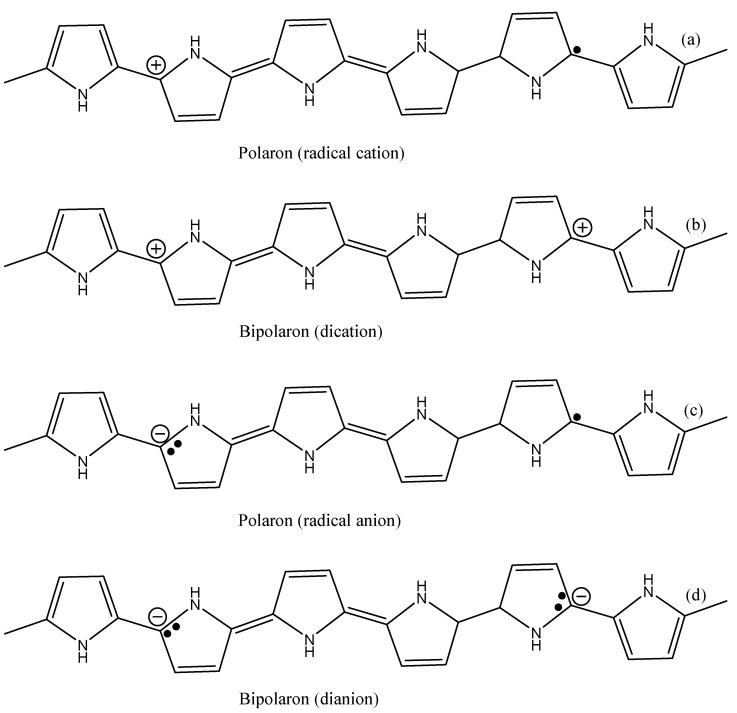
Chemical structures of charged positive/negative polaron and bipolaron in PPY. (**a**) Positive polaron, (**b**) positive bipolaron, (**c**) negative polaron, and (**d**) negative bipolaron.

As a continuous sequence following the formation of a charged soliton, a polaron is formed by virtue of the combination of the neighboring neutral conjugated polymer segment with the created charged soliton segment. The charge of the polaron depends on the type of doping, be it radical anions in n-type or radical cations in p-type doping. For instance, in p-type doping of polypyrrole (PPY), one electron is eliminated from the conjugated polymer chain, resulting in the formation of a radical cation, referred to as a positively charged polaron (see Scheme 3). Scheme 3 also depicts negatively charged polaron because of n-type doping, for comparisons sake. The created polaron state results in the formation of a new electronic state that is localized, situated in proximity to the valence band that consists of a single electron that is unpaired with a half spin (see Figure 1). For PPY, the new polaron states are known to reside at about 0.5 eV above the valence band edges [42,43]. 

The further oxidation of the already existing polaron, which is the elimination of the second electron from the chain that already contains the first created polaron, results in the formation of a bipolaron. This implies that, a dication species, referred to as a bipolaron, is formed by the combination of two positive polarons, resulting in a spinless defect state because of the paired electrons in opposite orientation (see Figure 1). For bipolarons, two newly created electronic states are situated inside the band gap, with each state consisting of a single electron that is unpaired. In comparison to polaron states, the higher state in bipolarons is more downshifted from the conduction band while the lower state is more upshifted from the valence band. In doped PPY, these bipolarons are situated at 0.75 eV [43]. For a heavily doped conjugated polymer, the two newly created bipolaron states found inside the band gap are capable of overlapping to generate a bipolaron band that can then act to a certain degree as a full band responsible for high electrical conductivity.

#### 2.2.1. P-Type Doping

**Scheme 4:**
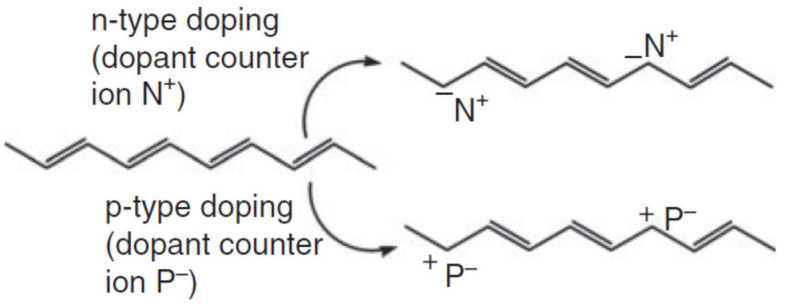
The n- and p-type doping procedure in conjugated polymers. Reproduced with permission from reference [41], copyright (2013), John Wiley and Sons.

Analogous to the concept of inorganic silicon semiconductor doping, where boron and phosphorus are the introduced foreign materials as p- and n-type dopants, respectively, dopants in conjugated polymers are similarly classified as p- and n-type. In the context of conjugated polymers, p-type doping is a process in which electrons are removed from the HOMO energy level of the conjugated polymer by utilizing oxidizing agents. In this case, the oxidizing agent accept electrons from the polymer, thereby being referred to as the acceptor, and leaves the conjugated polymer with a positive charge on the surface, as outlined and illustrated in Scheme 4. Due to the abundant π-excessive characteristic of conjugated polymers, numerous oxidizing agents have found ease and effectiveness in removing electrons from the conjugated polymer backbone. The list of the common p-type dopants classified according to the chemical nature such as organic, inorganic, and polymeric is tabulated in Table 1. Upon p-type doping, positively charged conjugated polymer chains and a counter anion are induced, resulting in the formation of an ionic complex.

#### 2.2.2. N-Type Doping

N-type doping is a process in which electrons are donated to the LUMO energy level of the conjugated polymer by utilizing reducing agents. In this case, the reducing agent donates electrons to the polymer, thereby being referred to as the donor, and leaves the conjugated polymer with a negative charge on the surface, as outlined and illustrated in Scheme 4. Upon n-type doping, negatively charged conjugated polymer chains and a counter cation are induced, resulting in the formation of an ionic complex. As opposed to p-type doping, n-type doping requires the use of strong reducing agents (e.g., alkali metals) since conjugated polymers in nature are already rich in π-electrons in their 2p_z_ orbitals. As a result, reports on n-type doping of conjugated polymers are narrowed and restricted. Therefore, the list of the common inorganic n-type dopants is tabulated in Table 2.

## 3. Synthesis Methods and Modifications of π-Conjugated Polymers

Due to the interesting optical and electrical properties exhibited by conjugated polymers, as discussed in Section 1 of this review, the scope of material design as per the requirement has led to an increase in the development of a variety of techniques for synthesis of conjugated polymers. Generally, the techniques to synthesize conjugated polymers are of three types: (1) oxidative polymerization, (2) condensation polymerizations, and (3) metal catalyzed cross coupling. 

### 3.1. Oxidative Polymerizations

**Scheme 5:**
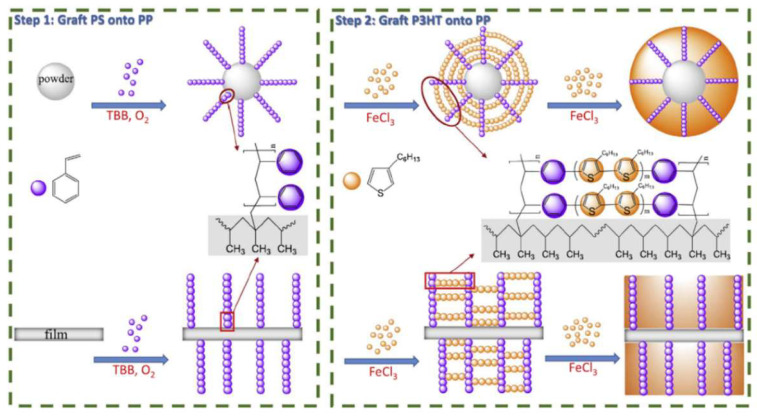
Grafting of polystyrene (PS) and poly(3-hexylthiophene) (P3HT) on polypropylene (PP). Reproduced with permission from reference [61], copyright (2018), Elsevier.

Among the numerous synthetic techniques for the construction of conjugated polymers, the most frequently utilized techniques are electrochemical oxidative polymerizations at electrode surfaces [56] and chemical oxidative polymerizations using oxidizing agents [57]. For chemical oxidative polymerization, a number of aromatic conjugated polymers such PTh, PPY, PEDOT, and their derivatives by incorporation of side alkyl chains such as P3HT using 3-hexyl as the side chain, have been synthesized using oxidizing agents such as FeCl_3_ and ammonium persulfate, (NH_4_)_2_S_2_O_8_ [58]. Most recently, the design of these polymers, through chemical oxidative polymerization, has far expanded and evolved from the known linear PThs and PPYs polymers, using monomers of these polymers to form either linear copolymers or giant branched macromolecules towards improving the conventional properties exhibited by the pristine conjugated polymers for the application of interest. For example, Ramoroka et al. [59] prepared a novel 2,3,4,5-tetrathienylthiophene-*co*-poly(3-hexylthiophene-2,5-diyl) (TTT-*co*-P3HT) donor polymer through chemical oxidative polymerization using FeCl_3_ as the oxidant in Chloroform. In this study, the repeating units of the monomer 3-hexylthiophene (3HT) grew out on the surface of 2,3,4,5-tetrathienylthiophene through α–α coupling of the aromatic thiophene rings between TTT and 3HT, which resulted in the formation of the novel polymer TTT-*co*-P3HT that found application in OSCs as a donor. In a similar fashion, Yonkue et al. [18] decorated poly(propylenethiophenoimine) dendrimer core with pendants of poly(3,4-ethylenedioxythiophene) that grew from the core, resulting in an electroconductive conjugated star copolymer, synthesized through chemical oxidative polymerization using (NH_4_)_2_S_2_O_8_ as the oxidant in a solvent mixture of chloroform and water. This poly(propylenethiopheneimine)-*co*-poly(3,4-ethylenedioxythiophene) (G1PPT-*co*-PEDOT) demonstrated improved optical, electrical, and photophysical properties, desirable properties that may be beneficial for photovoltaics and optoelectronic devices. Baleg et al. [60] modified poly(propylene imine) dendrimer by polymerizing pyrrole monomer units on the dendrimer principal core using (NH_4_)_2_S_2_O_8_ as an oxidizing agent in a solvent mixture of water and chloroform, which resulted in a conductive star copolymer. Hai et al. [61] modified polypropylene (PP) on the surface with P3HT via oxidative chemical polymerization using FeCl_3_ dispersed in hexane (see Scheme 5). The successful modification or grafting was achieved through two synthetic steps: (1) PP was first functionalized with styrene, yielding polystyrene-graft-polypropylene (Ps-g-PP), and (2) subsequent attachment of P3HT on the surface of Ps-g-PP through cross-linking of aromatic styrene and 3HT unit, enabled by the presence of the FeCl_3_ oxidant. The incorporation of P3HT on modifying PP resulted in the appearance of absorption peaks in the visible region, designating a formed overall π-conjugated system, and thus enhancing conductivity. 

**Scheme 6:**
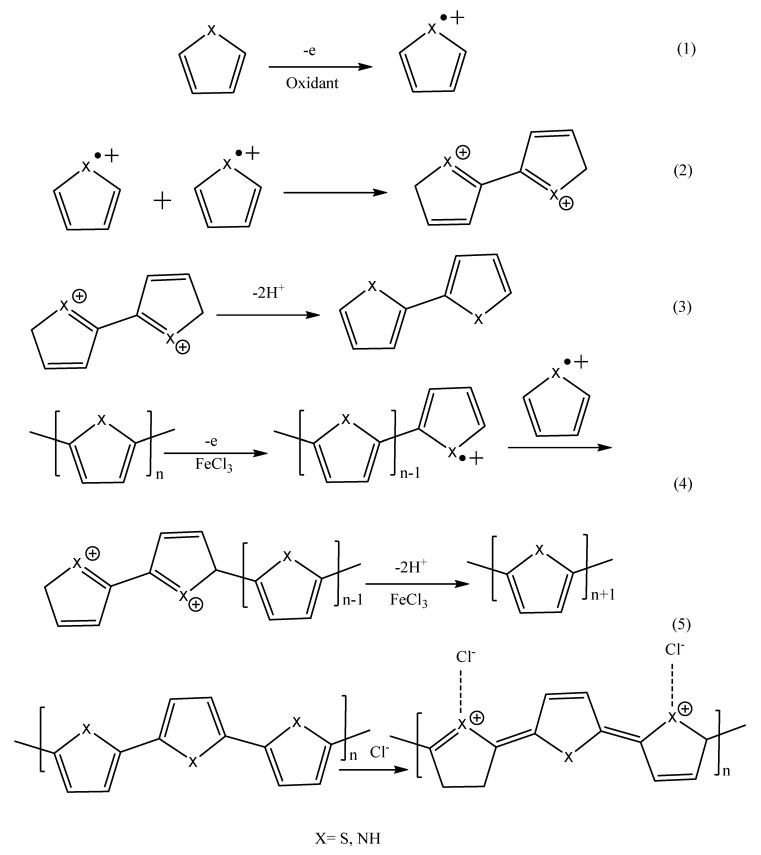
General mechanism of oxidative polymerization through 5 steps [63].

In another similar study, Hai et el. [62] modified polypropylene (PP) on the surface with P3HT via oxidative chemical polymerization using FeCl_3_ dispersed in hexane; however, this time, 3-(4-ethenylphenyl)thiophene was grafted in the first step instead of styrene, followed by cross-linking between 3-(4-ethenylphenyl)thiophene and 3HT through α–α coupling, resulting in P3HT-g-PP. Wang et al. [63] fabricated a conjugated conductive system by coating PEDOT layers on the surface of polypropylene-graft-poly(acrylic acid)(PP-g-PAA) for gas sensing, through in-situ oxidative polymerization using FeCl_3_. Moreover, Hai et al. [64] synthesized unmodified linear polymer chains of P3HT oxidatively using FeCl_3_ in various solvents, investigating the effect of molar ratio of oxidizer/3-hexylthiophene monomer. In addition, it was discovered that a high molecular weight polymer is easily obtained reliably in hexane and ethanol-free chloroform using a blend ratio that is less than 1 of the FeCl_3_/3HT oxidative polymerization [64]. Generally, it is reported that chemical oxidation polymerization using FeCl_3_ as the oxidant, usually produces excellent yields and high molecular weight polymers [65]. Research work undertaken on the chemical polymerization is summarized in Table 3. The polymerization mechanism for the formation of pyrrole and thiophene polymers is well-documented [66]. Scheme 6 illustrates the mechanism of thiophene-pyrrole oxidative polymerization. The mechanism involves the formation of monomer radical cation (step 1) because of oxidation induced by the introduction of the oxidant (FeCl_3_). Once oxidation has occurred, two radical cations combine via α–α coupling (step 2), followed by the formation of conjugation by deprotonation (step 3). From this point, polymerization continues from n-mer to (n + 1)-mer (step 4) until the undoped or doped state (step 5).

**Scheme 7:**
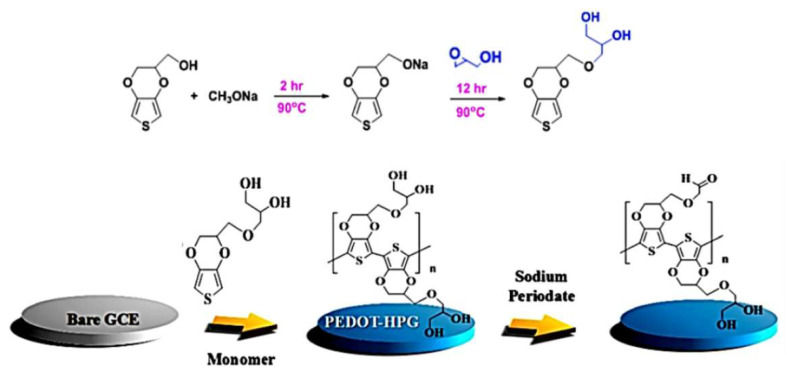
Synthesis of new EDOT-based monomer (top), and electrochemical oxidative polymerization of PEDOT-graft-HPG (bottom). Reproduced with permission from reference [68], copyright (2019), Elsevier.

On the other hand, in electrochemical oxidative polymerization, the conjugated polymer is prepared by a direct deposition of the polymer film on the surface of an electrode from a monomer solution that contains an electrolyte, using electrochemical techniques such as cyclic voltammetry [67]. An example is illustrated in Scheme 7, where Ma et al. [68] electrochemically synthesized PEDOT grafted with hyperbranched polyglycerol on the surface of a bare glassy carbon electrode (GCE). In contrast to chemical oxidative polymerization where a chemical oxidizing agent is used, an applied potential that is higher than the oxidation potential of the respective monomer to be polymerized, is required in the case of electrochemical oxidative polymerization in order to initiate polymerization. Therefore, this implies that, the electrochemical oxidative polymerization mechanism is identical to the chemical oxidative polymerization mechanism depicted in Scheme 6, except that an applied potential is used as the initiator instead of the FeCl_3_ oxidant. 

Numerous varieties of linear and branched conjugated polymers have been extensively and successfully synthesized using this route. For example, Cakal et al. [69] prepared an analogue of disubstituted 3,4-propylenedioxythiophenes, namely poly(3,3-bis(cyclohexylmethyl)-3,4-dihydro-2H-thione [3,4-b][1,4] dioxepin) (P_22_ProDOT-CycHex_2_), which was carried out in an electrolytic solution of 0.1 M tetrabutylammonium hexaflourophosphate (TBAH) dissolved in a mixture of acetonitrile (ACN) and dichloromethane (DCM). This polymer exhibited a faster response time (0.7 s) and a higher coloration efficiency (769 cm^2^/C) during oxidation, which showed a potential towards application in electrochromic devices. In a similar approach, another study, Cakal et al. [70] converted 5-(2-ethylhexyl)-1,3-di(thiophen-2-yl)-4H-thieno [3,4-c] pyrrole-4,6(5H)-dione (TTPT) monomer to its corresponding polymer PTTPT, carried out on the surface of a platinum electrode in an electrolytic solution of 0.1 M TBAH in a solvent mixture of acetonitrile (CAN) and dichloromethane (DCM). Baleg et al. [71] modified a bare platinum electrode by electrochemical attachment of generation 2 poly(propylene imine)-*co*-polypyrrole conducting dendrimeric star copolymer (G2PPI-*co*-PPY) using an electrolytic aqueous solution of 0.1 M LiClO_4_ containing pyrrole monomer and pyrrole-functionalized dendrimer (G2PPI-2Py). This conjugated dendrimeric star copolymer revealed relatively high ionic conductivity compared to linear PPY, facilitated by freely flowing charge between the holes generated by the arms of PPY on the surface of G2PPI-2Py. In another example, Olowu et al. [72] developed and fabricated an aptamer biosensor using an electrochemically synthesized conjugated system of generation 1 poly(propylene thiophenoimine)-*co*-polythiophene (G1PPT-*co*-PEDOT) dendritic star copolymer modified in bare gold (Au) electrode in an aqueous electrolytic solution consisting of 0.1 M LiClO_4_ and sodium dodecyl sulphate. Moreover, Makelane et al. [73] followed a similar approach and fabricated a phenanthrene sensor using a conjugated system of generation 3 poly(propylene thiophenoimine)-*co*-poly(3-hexylthiophene) (G3PPT-*co*-P3HT) star copolymer modified on Au electrode in an electrolytic solution of 0.1 M Bu_4_NClO_4_ dissolved in ACN containing 3HT monomer.

### 3.2. Metal-Catalyzed Cross-Coupling Reactions

Among the numerous varieties of synthetic techniques for organic compounds and conjugated polymers, cross-coupling reactions stand amongst the most important in chemistry. These reactions are a highly valuable powerful tool employed to prepare a diverse variety of organic compounds, from synthetic and natural bioactive compounds to new organic compounds and conjugated polymers, in all fields of chemistry [74]. These are reactions involving the reaction between an organometallic reagent (Ar^1^–M) with an unsaturated organic halide (Ar^2^–X) or pseudohalide using transition metal complexes, such as palladium and nickel as catalysts for C(sp^2^)–C(sp^2^) hybridized bond formation [74,75,76,77]. The well-known, mostly used, and recognized cross-coupling polymerization methods are the Suzuki–Miyaura, Stille, and Kumada using nucleophilic reagents such as organoboron, organotin, and aryl Grignard reagents, respectively. In contrast to polymers formed by oxidative polymerizations, the conjugated polymers formed using cross-coupling reactions are in their undoped neutral state. 

#### 3.2.1. Suzuki–Miyaura Cross-Coupling Reaction

**Scheme 8:**
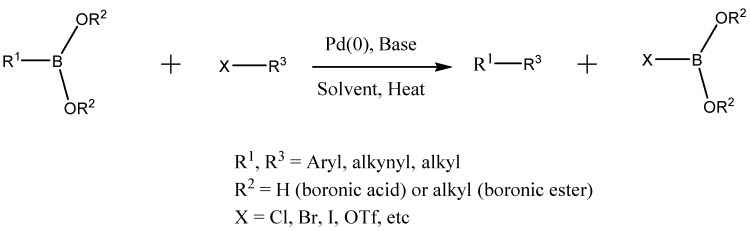
General Suzuki–Miyaura cross-coupling reaction.

Amongst the cross-coupling reactions, the Suzuki–Miyaura has become the most extensively applied and versatile transition metal catalyzing technique for the construction of a variety of conjugated polymers and organic compounds through the formation of C(sp^2^)–C(sp^2^) bonds [78,79]. This reaction involves coupling between aryl boronate species (eg. Arly boronic acid and aryl boronate esters) with an aryl halide in the presence of palladium, Pd (0), as a catalyst, and base for the activation of the boron compound (Scheme 8) [80,81,82]. The success and versatility of this reaction is owing to the commercial availability of boronic acids, which are non-toxic and environmentally friendly, compared to organostannane and organozinc reagents employed in Stille and Negishi coupling [83]. In addition, the reaction has a high degree of functional group tolerance, works under relatively mild conditions, and the reagents used can be easily prepared and are cheap [83]. As such, the Suzuki–Miyaura cross coupling has found utility in synthesizing substituted conjugated biphenyls, poly-olefins, styrenes, and in large scale industrial applications.

**Scheme 9:**
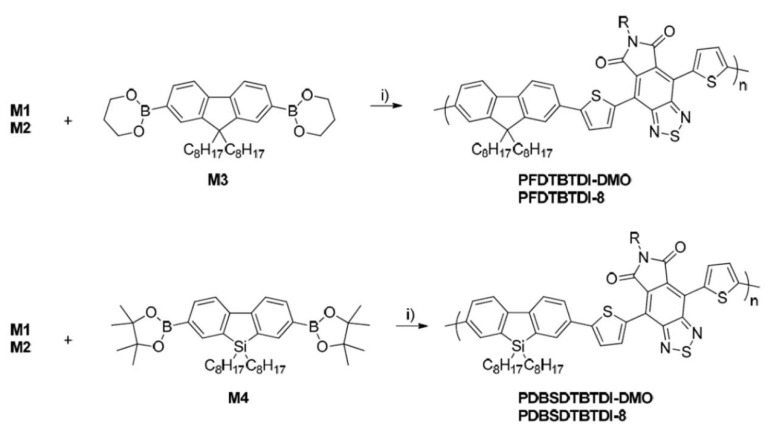
Synthesis of (**i**) PFDTBTDI-DMO, PFDTBTDI-8, PDBSDTBTDI-DMO, and (PDBSDTBTDI-8 via Suzuki polymerization. Reagents and conditions (**i**) Pd(OAc)_2_/P-(o-tol)_3_, NaHCO_3_, anhydrous THF, 90 °C, 21–30 h [87].

Since the discovery and inception of the Suzuki–Miyaura concept, in 1979, where Miyaura et al. [84], demonstrated that the conjugated (E)-dienes or (E)-enyes with high regio- and stereo-specificity in good yields are obtainable via hydroboration of 1-alkynes reacting with 1-alkenyl halides or alkynyl halides in the presence of tetrakis(triphenylphosphine) palladium and base, a number of conjugated polymers have been prepared, now referred to as Suzuki Polycondensation (SPC). SPC is a step-growth polymerization technique, a derivative of the Suzuki–Miyaura reaction, in which a continuous sequence of Suzuki couplings occur between the monomers, and subsequently form a polymer. SPC is divided into two approaches: (1) AA/BB approach-two monomers are used as the starting reagents, in which the first monomer is substituted with two boronic acid functional groups and the second monomer is carrying two halides, to form an alternating copolymer [85]; (2) AB approach- uses one monomer substituted with both a boronic acid and halide in order to form a homo-polymer [83]. The AA/BB approach is of the utmost significance in altering the properties exhibited by pristine conjugated polymers through preparation of alternating donor-acceptor copolymers that are extensively applied in optoelectronic devices. For example, Lee et al. [86] synthesized a benzothiadiazole- based conjugated copolymer by reacting 4,7-dibromobenzo[c]-1,2,5-thiadiazone with 1,3,5-phenyltriboronic acid in the presence of tetrakis(triphenyl phosphine) palladium (0) catalyst and potassium carbonate as base in dimethyl formamide (DMF). The authors investigated the catalytic properties of the benzothiadiazole copolymer and concluded that it can be applied in water treatment and organic synthesis reactions using sunlight as an energy injection source. Moreover, the copolymer exhibited enhanced and broadened visible light absorption, which is a desirable property for conjugated polymers applied in OSCs. Murad et al. [87] prepared four alternating donor-acceptor copolymers (Scheme 9), PFDTBTDI-DMO, PDBSDTBTDI-8, PFDTBTDI-8, and PDBSDTBTDI-DMO for photovoltaic application through Suzuki polymerization by reacting bis-borate esters with dibromides, performed using Pd(OAc)_2_/P(o-tol)_2_ catalyst and NaHCO_3_ in anhydrous THF. Low-lying HOMO energy levels of about −5.59 eV were realized when using these polymers as electron-donating materials, which are beneficial for the chemical stability of the polymers in oxygen and should subsequently lead to higher open-circuit voltage (*Voc*) values, thus improving the OSC performance.

In another study, Vazquez-Guillo et al. [88] prepared conjugated aniline-fluorene alternate copolymers through microwave-assisted Suzuki polymerization. Briefly, the copolymer poly[1,4-(2/3-aminobenzene)-*co*-alt-2,7-(9,9′-dehexylfluorene)] (PAF) as a molecular model with solvent sensing property was realized by mixing 2,5-dibromoaniline and 9,9-dihexylfluorene-2,7-diboronic acid bis(1,3-propanediol) ester in the presence of Pd(0) and potassium carbonate as base dissolved in a solvent mixture of THF/H_2_O placed in the microwave. By this method, the products were acquired in shorter periods as compared to traditional synthetic methods, which gave average yields and moderate molecular weight copolymers. The authors envisaged that these copolymers could find applications in sensing.

**Scheme 10:**
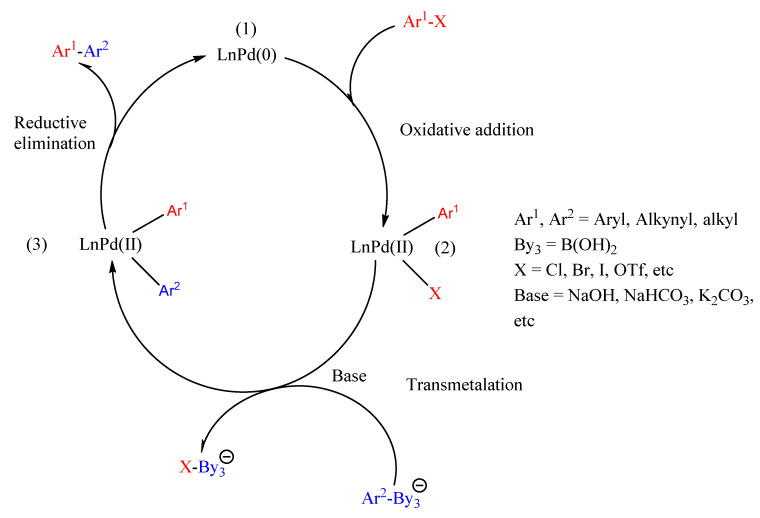
The catalytic cycle of the Suzuki–Miyauya cross-coupling reaction.

The general mechanism of Suzuki–Miyaura follows a catalytic cycle that is similar to the other metal-catalyzed cross coupling reactions, involving three primary steps, in a sequential manner, namely: (1) oxidative addition, (2) transmetalation and (3) reductive elimination. However, reports have shown that the transmetalation step or the activation of boron reagent makes Suzuki–Miyaura coupling different from the other metal-catalyzed cross coupling processes [81]. This is because, in the Suzuki–Miyaura coupling transmetalation step, sodium hydroxide or potassium carbonate as the base is required [89]. In the mechanism (Scheme 10), the first step involves the oxidation of the palladium catalyst from LnPd(0) to LnPd(II) through the coupling of aryl halide (Ar^1^X) with the palladium catalyst (LnPd(O)) to form an organopalladium complex Ar^1^LnPd(II)X, where the Ar^1^-X bond is broken and the palladium sandwiches itself in between the Ar^1^ and X. This step is often deemed as the rate determining step. The second step is the transmetalation step, where an organometallic reaction takes place and a ligand transfer occurs from the incoming organoboron specimen (Ar^2^-By_3_^−^) to the oxidized palladium complex (Ar^1^LnPd(II)X). In this step, a base is required to react with the organoboron specimen, creating a negative charge boronic species, which is highly nucleophilic and ready to undertake ligand transfer with the palladium complex (metathetic exchange), forming another palladium complex, Ar^1^LnPd(II)Ar^2^. The reductive elimination is the final step in the catalytic cycle, where the Ar^1^LnPd(II)Ar^2^ palladium complex proceeds to eliminate the required product (Ar^1^-Ar^2^) and is converted back into Pd(0).

#### 3.2.2. Stille Cross-Coupling Reaction

**Scheme 11:**
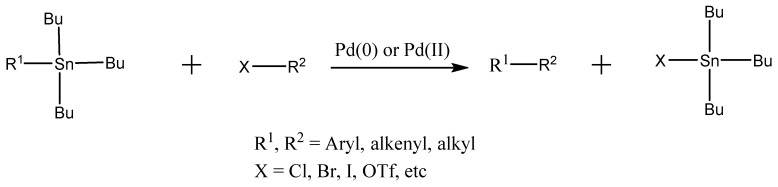
General Stille cross-coupling reaction.

Similar to the Suzuki–Miyaura cross coupling, the Stille cross coupling has also found extensive application and versatility in transition metal-catalyzed reactions for the construction of a variety of conjugated polymers and organic compounds through the formation of C(sp^2^)–C(sp^2^) bonds [90]. This reaction involves coupling between aryl stanners species with organic electrophiles such as aryl halide in the presence of palladium, Pd (0), as a catalyst (Scheme 11) [91,92]. The success and widespread use of this reaction is attributed to the mild reagents of organotin, which are well matched with a diverse number of functional groups [93,94]. Moreover, organostanners are insensitive to moisture and oxygen, allowing stille reactions to be undemanding and performed under relatively neutral conditions [93,94]. As good as this reaction is, the drawback lies with the contamination and toxic waste products as a result of the use of organotin compounds such as Bu_3_SnR [95,96,97].

**Scheme 12:**
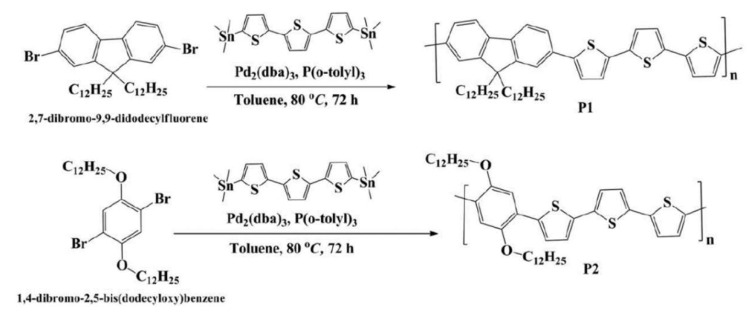
Synthesis of P1 and P2 using Stille polycondensation reaction. Reproduced with permission from reference [106], copyright (2015), John Wiley and Sons.

Since the discovery of the Stille coupling concept after the efforts made by Migita et al. [98], in 1978, where Still et al. [99], demonstrated the coupling of a variety of alkyl tin reagents with numerous acyl and aryl halides under mild conditions with much improved better yields in the presence of a palladium catalyst, a number of conjugated polymers have been prepared, now referred to as Stille polycondensation (SP). Much as Suzuki polycondensation, the Stille polycondensation is a step-growth polymerization procedure, where electron-rich distannane monomers are reacted with electron-deficient organodihalide reagents in the presence of palladium catalyst. This reaction is known to produce high molecular weight polymers with excellent yields, and as a result, SP has been extensively applied in the construction of various π-conjugated polymers [100,101,102], which has led to advanced technologies for different applications such as polymer solar cells [103], polymer light-emitting diodes [104], and polymer field-effect transistors [105]. For example, Hanif et al. [106] prepared two conjugated polymers, P_1_ and P_2_, by employing the Stille polycondensation (Scheme 12), reacting 2,7-dibromo-9,9-didodecylfluorene and 1,4-dibromo-2,5-bis(dodecyloxy) benzene with 5,5”-bis(trimethylstannyl)-2,2′-:5′-2”-terthiophene in the presence of Pd catalyst for photovoltaic application. The polymers exhibited good higher molecular weight, good stability, well defined UV-Vis, and lower band gaps as required in PV applications.

Yin et al. [107] synthesized a series of boron-containing conjugated polymers, PB2T and PFB2T, with boron atoms incorporated into the conjugated polythiophene main chain via Pd-catalyzed coupling of stannylated thienylborane monomers. The authors stressed that these boron-containing conjugated polymers call for a broad adaptation in the development of novel optoelectronic materials, as rendered by the excellent long-term chemical stability to moisture and air, facile tunability, and the excellent fluorescence properties. Kim et al. [108] prepared a benzodithiophene-based donor-acceptor polymer (PTB7) via Stille polymerization using both thermal and microwave as a comparative study for solar cell application. In this study, it was found that the microwave assisted Stille polymerization of PTB7 gave higher molecular weight polymer compared to thermal heating; however, the power conversion efficiency of the resultant solar cell was lower than that of thermal PTB7. This was believed to be due to structural defects generated by microwave, which consequently resulted in the decrease of the short-circuit current density (*J_sc_*) and fill factor (*FF*).

**Scheme 13:**
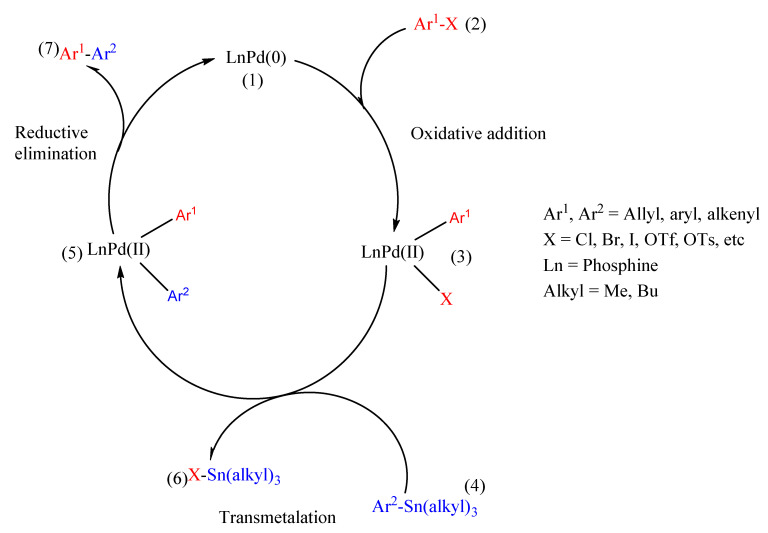
The catalytic cycle of the Stille cross-coupling reaction.

The general mechanism of Stille cross coupling (Scheme 13) is similar to that of Suzuki–Miyaura, which follows a catalytic cycle involving three primary steps, in a sequential manner, namely: (1) oxidative addition (2) transmetalation and (3) reductive elimination.

In the mechanism, the first step involves the oxidation of the palladium catalyst from LnPd(0) to LnPd(II) through the coupling of aryl halide (Ar^1^X) with the palladium catalyst (LnPd(0)) to form an organopalladium complex Ar^1^LnPd(II)X, where the Ar^1^-X bond is broken and the palladium sandwiches itself in between the Ar^1^ and X. This step is often deemed as the rate determining step. The second step is the transmetalation step, where an organometallic reaction takes place and a ligand transfer occurs from the incoming organotin specimen (Ar^2^-Sn(alkyl)_3_) to the oxidized palladium complex (Ar^1^LnPd(II)X). The reductive elimination is the final step in the catalytic cycle, where the Ar^1^LnPd(II)Ar^2^ palladium complex proceeds to eliminate the required product (Ar^1^-Ar^2^) and is converted back into Pd(0).

#### 3.2.3. Kumada Cross Coupling

**Scheme 14:**
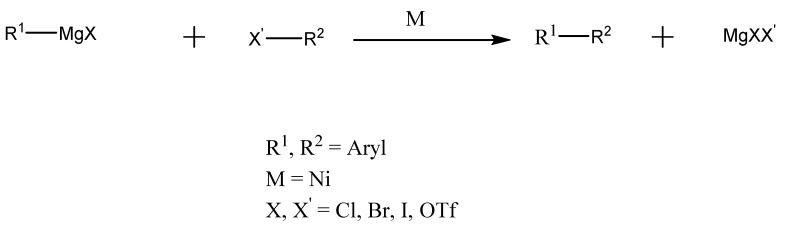
General Kumada cross-coupling reaction.

In addition to Suzuki and Stille cross couplings, Kumada–Corriu coupling has also been widely used as a synthetic technique for the construction of conjugated polymers and other organic compounds through the formation of C(sp^2^)-C(sp^2^) bonds [109]. This type of reaction involves the coupling between Grignard reagents and aryl halides, facilitated by nickel complexes as catalysts [109] (Scheme 14). Its wide range of practical applicability is owing to reasons such as simple procedures, high yields, mild reaction conditions, and the high purity of the coupling products obtained [110]. In addition, because of the use of Grignard reagents, which are highly reactive, the coupling has very limited functional group tolerance. As such, Kumada coupling has been employed in the synthesis of polyalkylthiophenes which are useful in organic electronic devices, and large-scale industrial production of aliskiren, a hypertension medication.

**Scheme 15:**
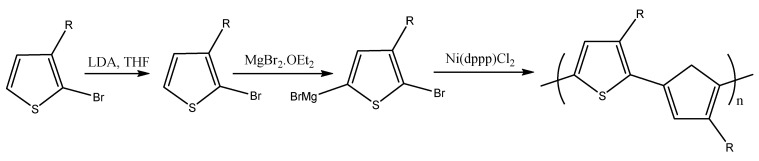
Synthesis of poly(3-alkylthiophenes) (P3AT) [112].

Since the discovery of the Kumada coupling in 1972, where Kumada et al. [111] demonstrated a useful preparative method for synthesizing unsaturated organic compounds—which involved selective cross coupling between a Grignard reagent and an aryl halide catalyzed by a nickel-phosphine complex, which has produced high product yields—the coupling method gained wide spread attention for preparation of conjugated polymers that have a variety of potential applications in organic solar cells and light-emitting diodes. In 1992, McCullough and Lowe et al. [112] synthesized the first series of regioselective poly(3-alkylthiophenes) (Scheme 15) from 2-bromo-3-alkylthiophenes through the use of a nickel-catalyzed cross-coupling reaction, which produced yields of between 20–60% and exhibited high electrical conductivities, desirable for applications in opto-electronic devices. Since this initial preparation, the synthesis has been modified to obtain high yields and large numbers of conjugated polymers to find applications in organic electronic devices.

For example, Xie et al. [113], prepared two regioregular polythiophene derivatives for application in organic solar cells, P3SHT and PTST with incorporation of thioalkyl chains, through Kumada catalyst-transfer polycondensation (KCTP). In comparison to the traditionally used P3HT polymer with hexyl chains, P3SHT and PTST exhibited stronger absorption and downshifted energy levels, which are properties desirable for OSCs. Lee et al. [114] prepared a conjugated block copolymer (P3HT-b-PFTBT) to be used as an active-layer material in OSC, through a combination of Kumada catalyst transfer/Grignard metathesis and Suzuki polycondensation. The block copolymer formation proceeded through two synthetic steps: (1) synthesis of P3HT using Kumada coupling (2) followed by the addition of PFTBT on P3HT as an extension using Suzuki polycondensation. The resultant block copolymer demonstrated enhanced microphase separation, minimal homopolymer impurities, and higher power conversion solar cell efficiencies when used as the active layer in the devices.

**Scheme 16:**
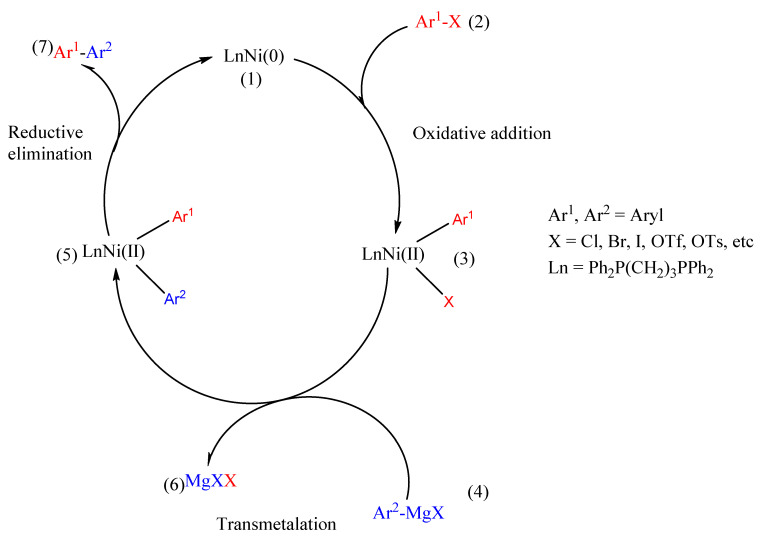
The catalytic cycle of the Kumada cross coupling reaction.

Similar to the Suzuki and Stille cross couplings, the Kumada coupling mechanism (Scheme 16) follows a catalytic cycle involving three fundamental steps, namely: (1) oxidative addition (2) transmetalation and (3) reductive elimination.

In the mechanism, the first step involves the oxidation of the active nickel catalyst from LnNi(0) to LnNi(II) through the coupling of aryl halide (Ar^1^X) with the nickel catalyst (LnNi(0)) to form an organonickel complex Ar^1^LnNi(II)X, where the Ar^1^-X bond is broken and the nickel sandwiches itself in between the Ar^1^ and X. The second step is the transmetalation step, where an organometallic reaction takes place and a ligand transfer occurs from the incoming Grignard reagent (Ar^2^MgX) to the oxidized nickel complex (Ar^1^LnNi(II)X) to generate a diarly nickel (II) complex. The reductive elimination is the final step in the catalytic cycle, where the Ar^1^LnNi(II)Ar^2^ nickel complex proceeds to eliminate the required product (Ar^1^-Ar^2^) and is converted back into Ni(0).

## 4. *π*-Conjugated Polymers in Solar Cell Applications

π-Conjugated polymers have gained increased scientific attention due to their low production cost, lessened complexity in the fabrication processes, and flexibility. In addition, the increased attention is also owing to their superior advantages such as the ability to tailor their electrical and optical properties by use of structural modifications, ease of synthesis, and ease of processability [19]. Therefore, these advantageous properties have rendered extensive usage of the polymers in the broad scope of solar cell technology as compared to inorganic materials. They have found extensive use in organic solar cells, perovskite solar cells, dye-sensitized solar cells, and silicon solar cells.

### 4.1. General Working Principle of Solar Cells and Their Characteristics

**Figure 2:**
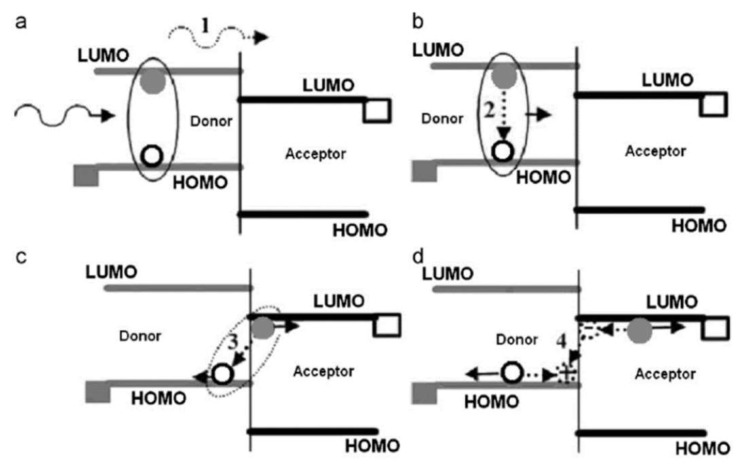
General operating principle of a photovoltaic cell. The entire process is outlined as follows: (**a**) photon absorption generates excitons, (**b**) excitons diffuse to the heterojunction, (**c**) excitons dissociate into charge carriers, and (**d**) carriers are transported to the electrodes for collection. The diagrams also depict other loss mechanisms as follows: (1) non-absorbed photons, (2) exciton decays, (3) geminate recombination of the bound pair and (4) bimolecular recombination. Reproduced with permission from reference [116], copyright (2013), Elsevier.

A French scientist, Edmund Becquerel, first discovered the photovoltaic phenomenon in 1839 using an electrolytic cell, where he observed that exposing certain materials to visible light could create a weak electrical current [3,115]. It was noted that, for the phenomenon of photovoltaic to be effective and operate efficiently, the semiconducting material must be able to absorb a sufficient quantity of incident photons, which generate and promote electrons to higher energy levels (conduction band, CB), and carry an electrical field that increases the rate in which the excited electrons are transported in one specific direction, causing an induced electrical current [3,115]. Since then, the general working mechanism has been well studied and understood. The mechanism is believed to proceed through five operational steps (Figure 2) as follows:

- Generation of excitons due to absorption of sufficient number of photons by the semiconductor or absorbing materials that form a p-n junction;
- Diffusion of the generated excitons to the active interface of the p-n junction;
- Subsequent separation or dissociation of the photo-generated excitons into electrons and holes, referred to as charge carriers, at the junction;
- Transportation of the charge carriers using appropriate hole and electron transporting materials, commonly known as HTLs and ETLs;
- Lastly, collection of the charge carriers at the terminals of the junction, by anodic and cathodic electrodes, thereby resulting in electrical energy creation and flow.

**Figure 3:**
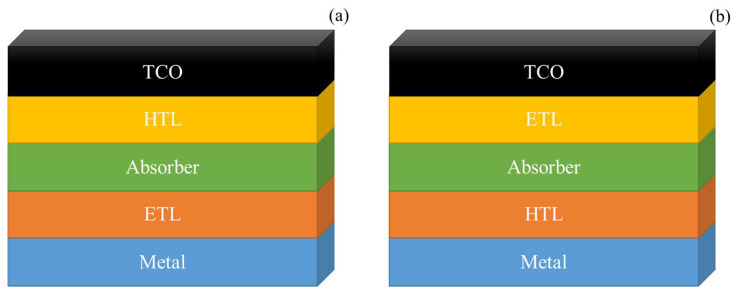
A typical general solar cell structure (**a**) conventional and (**b**) inverted.

A generalized conventional and inverted working solar cell structure of a device is depicted in Figure 3. As seen, it consists of several layers for different functions, namely TCO, HTL, absorber, ETL, and a metal. The TCO is a transparent conducting oxide, such as fluorene-doped tin oxide (FTO) and indium tin oxide (ITO), used as an anti-reflective coater (ARC) as well as the conducting anodic electrode on the front side of the cell. Usually, very thin films of these TCO layers of between 70–150 nm in silicon-based solar cells are required as to allow more transmission of light to the absorbing material and minimize optical losses. Studies conducted have shown transmittances of more than 90–95% with minimal sheet resistances and smooth surface morphologies when the TCO layer, particularly ITO, is between 80–150 nm [117,118,119]. Metallization of Au, Al, or Ag as metal contacts occur at the rear/back side of the cell. Studies conducted by Gwamuri et al. [120] have shown that an ITO thickness of 50 nm has transmittance of around 80% with higher sheet resistance values, not satisfactory for solar cell applications. Therefore, this affirms the threshold minimum of 70 nm in order to find a balance between transmittance, sheet resistivity, and smooth morphologies.

**Figure 4:**
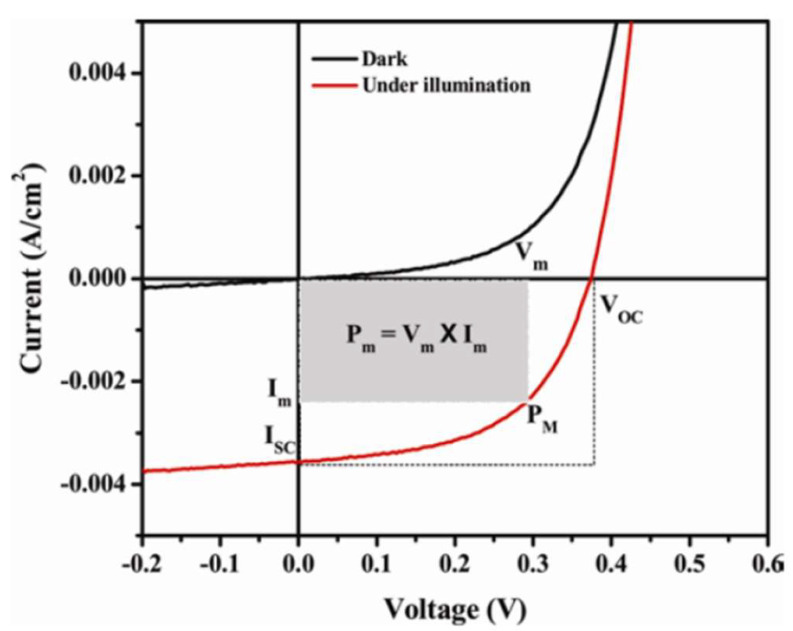
A solar cell device’s typical current−voltage curve. Reproduced with permission from reference [121], copyright (2013), Taylor & Francis Online.

The important parameters associated with determining and elucidating the performance of a solar cell are the fill factor (*FF*), open-circuit voltage (*V_oc_*), the short-circuit current density (*J_sc_*), and the power conversion efficiency (PCE). The typical current-voltage (I-V) curve is presented in Figure 4. The *FF* is defined as the ratio between the cell’s maximum power (Pm) and the product of the *V_oc_* and *J_sc_* (*V_oc_* × *J_sc_*). Under illumination, the *V_oc_* is characterized as the potential difference between the cell’s terminals at zero current flow through the terminals. *J_sc_* is the current produced by the cell under illumination when there is no other source of potential or external potential. The PCE is the ratio of the solar cell’s overall output power to the incident radiant power.

### 4.2. Conjugated Polymers as Emitters/HTLs in Organic-Silicon Hybrid Heterojunction Solar Cells

Hybrid heterojunction solar cells (SHJ) based on crystalline silicon substrates (c-Si) are drawing increased recognition owing to their diverse technological advantages [122]. The attention gained is owing to the use of hydrogenated amorphous silicon (a-Si:H), which can be deposited as either p-or n-type and intrinsic using plasma enhanced chemical vapour deposition (PECVD), leading to SHJ solar efficiency of 24.7% for rear or front contact structure and 25.5% for interdigitated rear contact architecture [123,124,125]. P-type a-Si:H is often used at the emitter region in a front contact structure working as both the emitter layer and HTL. As impressive as these SHJs are, their practical use is limited by the complexity in fabrication processes, high deposition temperatures of (*p*)-a-Si:H, and the expenses of the state-of-the-art PECVD method, which increases costs [126]. Additionally, the well-known absorption inability of blue light by a-Si:H on the front side [7,126,127] and the large resistivity at the ITO/a-Si:H interface which results in electrical losses [128], hinders the development of SHJs incorporating (*p*)-a-Si:H. Ideally, a very thin emitter layer should have low light absorption, low resistivity, and high conductivity. Alternatives such as using p-doped microcrystalline silicon thin-film emitter (*p*-(μc-Si:H) [129] and p-type silicon carbon (*p*-(a-SiC:H) emitter layer [126] in place of (*p*) a-Si:H have been developed but still lack the cutting edge due to the deposition of the layers using the complex and expensive PECVD technique. Therefore, looking for an efficient HTL or emitter is imperative and significant in electron-hole separation, transmission of light, and ultimately the excellent performance of the device.

**Figure 5:**
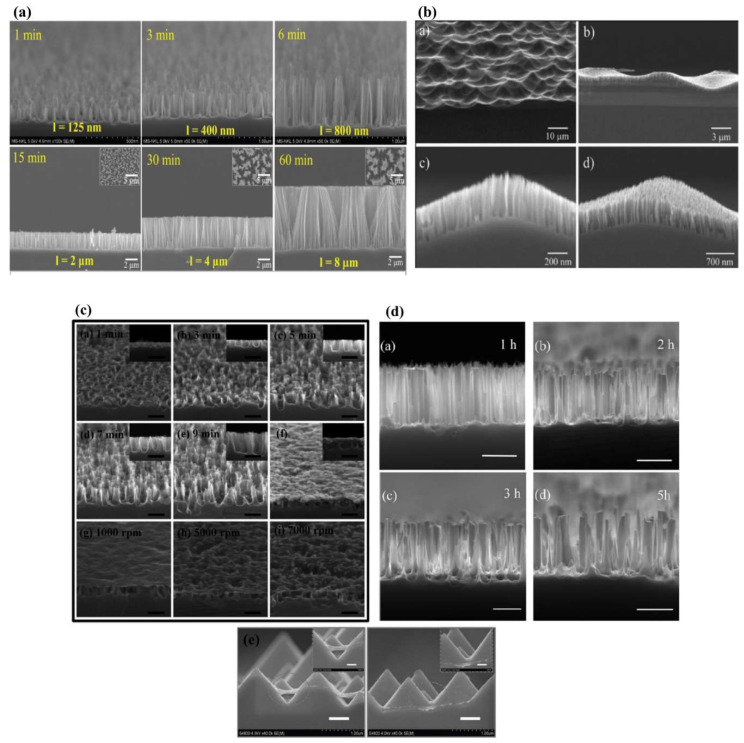
Comparisons of the cross-sectional area SEM images of the different morphologies of Si/PEDOT:PSS films. (**a**) Silicon nanowires (SiNWs) prepared by chemical etching with different etching time [145], (**b**) Silicon nanoholes (SiNHs) with micro-deserted structures (Reproduced with permission from reference [142], copyright (2014), Royal Society of Chemistry), (**c**) Silicon nanocones (SiNCs) at different etching times (Reproduced with permission from reference [146], copyright (2015), Royal Society of Chemistry), (**d**) Silicon nanopillar arrays (SiNPs) at different etching times (Reproduced with permission from reference [147], copyright (2012), Royal Society of Chemistry), and (**e**) Textured-Si with and without phthalic acid ester (Reproduced with permission from reference [143], copyright (2017), Wiley & Sons).

**Figure 6:**
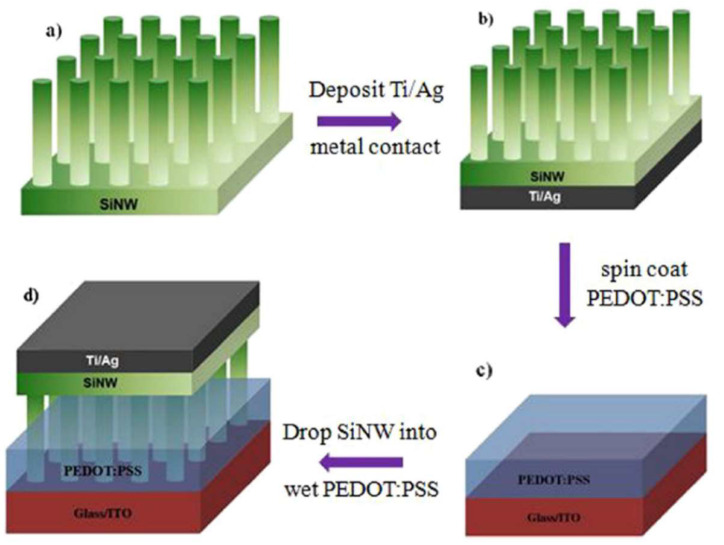
Schematic illustration of the fabrication process of organic-silicon hybrid heterojunction solar cell. (**a**) Fabricated Silicon Nanowires (SiNWs), (**b**) deposited Ti and Ag on the rear side of the Si wafer as cathode material, (**c**) Spin-coated PEDOT:PSS on ITO/glass substrate, and (**d**) immersion of SiNWs into wet PEDOT:PSS film. Reproduced with permission from reference [141], copyright (2015), AIP Publishing.

In order to lower the manufacturing cost of conventional silicon heterojunction solar cells, hybrid solar cells based on the blend of silicon semiconductors and organic materials are presently under investigation for future alternative photovoltaic devices due to their easy fabrication processes and low production costs [130,131]. Therefore, the use of conjugated conductive polymers offers an advanced feasible and delicate approach because of the likelihood of employing cost-effective and low temperature deposition techniques such as spin coating [132], ink-jet printing [133] and spray-pyrolysis [134]. Moreover, the presence of the semiconductor impedes the head-on attachment between the silicon base semiconductor and the metal anode, thereby decreasing carrier recombination at the front due to the charge carrier-blocking characteristics of the polymeric material as compared to the conventional approach [135,136]. Generally, a transparent conductive conjugated polymer is utilized as the emitter or HTL for lowering the fabrication cost without compromising the performance of the solar cells. In this regard, amongst the many p-type conjugated polymers, PEDOT:PSS has found more application as HTL layer in hybrid organic-silicon heterojunction solar cells. This is due to its good film forming properties, excellent transparency, low processing temperature, and high work function (~5.2 eV), which render it an effective HTL [137,138,139]. Therefore, the conjugated polymer is used to replace the (*p*) a-Si:H layer in the a-Si/c-Si heterostructure solar cell. For this technology, various forms of structured and planar silicon substrates (poly-Si, c-Si, Si, and μc-Si nanostructures in different sizes) are used as absorber materials. Uma et al. [140] fabricated the device with ITO/PEDOT:PSS-GO/SiNWs/(*n*) c-Si/Ti/Ag architecture and obtained an efficiency of 9.57%. The performance of the device was owing to the incorporation of 30% graphene oxide (GO) to form a blend mixture of PEDOT:PSS-GO, promoting high hole mobility to the ITO before carriers could recombine. Mahato et al. [141] obtained an improved efficiency of 11% by fabricating the solar cell structure of Ag/PEDOT:PSS-5% DMSO/(*i*) a-Si:H/(*n*) c-Si/(*i*) a-Si:H/(n^+^) a-Si:H/a-SiC:H/Ti/Al. The improved efficiency was ascribed to the fast hole mobility of the PEDOT:PSS-DMSO blend and the surface passivation using intrinsic hydrogenated amorphous silicon ((*i*)a-Si:H) at the PEDOT:PSS/(*n*) c-Si interface and (*n*) c-Si/(n^+^) a-Si:H interface, thereby reducing carrier recombination. Thiyagu et al. [142] obtained a 12% efficiency with a hierarchical surface composed of silicon nanoholes (SNHs) and micro-desert textures. The hierarchical surface provided excellent light harvesting due to internal multiple reflections. The morphology of the Si surfaces or Si nanostructures have a direct impact on the performance of the solar cells. These morphologies can be controlled by etching masks and deposition times in order to realize higher efficiencies. He et al. [143] fabricated a conventional Si/PEDOT:PSS structure on a textured-Si with pyramids. The problem associated with this approach is that the Si-pyramids cannot be fully coated by the PEDOT:PSS, leading to poor contact properties and inferior *Voc*. This problem was avoided by spin-coating a water-insoluble phthalic acid ester having a low viscosity on the PEDOT:PSS side of the cell, which improved the tensile stress of the PEDOT:PSS to have tight contact and full coverage with textured-Si. This led to the cell producing an efficiency of 16.2%. Ding et al. [144] constructed hybrid organic/inorganic solar cells based on a blend of silicon nanocrystals (NCs) and conjugated polymers and carefully optimized the devices through tuning the surface termination of Si NCs and selecting an appropriate conjugated polymer with a narrow band gap. Devices constructed with Si NCs terminated with chlorine exhibited reduced performance due to excessive carriers as a result of the high electronegativity of the halogen at the surface, which polarizes the electron density away from the Si NC core. The devices showed an improved in performance from 0.52% to 2.25% in PCE when Si NCs were terminated with hydrogen by treatment with HF vapor and using PTB7 as the conjugated polymer with narrow band gap as opposed to P3HT. The increased performance was attributed to the reduction in excessive carriers and the ability of PTB7 to harness light over a wider range than P3HT. The different morphologies of the Si substrates and Si nanostructures with PEDOT:PSS interactions are illustrated in Figure 5 and the typical fabrication process of the hybrid organic-silicon heterojunction is schematically depicted in Figure 6.

Despite the tremendous progress made by the hybrid cells, the stability of the PEDOT:PSS or p-type polymer based solar cell still provides a daunting task due to the hydrophilic and acid characteristics of the PEDOT:PSS [148,149]. Moreover, reports have emerged where PEDOT:PSS corrodes substrates, and more particularly ITO [150]. In addition, the hygroscopic properties of PEDOT:PSS [151], together with the chemical degradation of metal electrodes [152], results in the degradation of the solar cells. The stability issue was well demonstrated by Zellmeier et al. [153] where a 50% loss of the initial efficiency was realized after 1 day using the solar cell structure of Au/MoO_3_/P3HT/CH_3_-Monolayer/(*n*) c-Si/(*i*) a-Si:H/(n^+^) a-Si:H/Metal. In order to solve these challenges, inverted back contact device structures and thicker metal contacts are recommended as they are more mechanically stable and can successfully prevent photo-oxidation [153].

Zielke et al. [154] fabricated an inverted back junction organic-silicon solar cell by placing the PEDOT:PSS layer on the rear side instead of the front side which avoided parasitic light absorption and enabled an improved surface passivation. This solar cell with architecture Al/SiNx/Al_2_O_3_/n^+^-FSF/(*n*) c-Si/SiOx/PEDOT:PSS/Ag eventually achieved an efficiency of 17.4%. Moreover, Gogolin et al. [155] expanded on this concept and fabricated a hybrid solar cell using a p-type silicon absorber, an excellent passivation electron selective layer (a-Si:H (i/n) on the front side, and PEDOT:PSS as the HTL on the rear side and attained an efficiency of 16.2% with a record breaking *V_oc_* of 688 mV. To date, Schdmt et al. [156,157] have attained the record efficiency of 20.6% by applying an optimized p-type silicon surface pre-treatment. These results demonstrate that conductive p-type polymers are promising hole transfer materials in silicon heterojunction solar cells, substituting the boron-doped hydrogenated amorphous silicon ((*p*) a-Si:H) in order to reduce fabrication complexities and minimize production cost. More research results undertaken in this field are summarized in Table 4.

### 4.3. π-Conjugated Polymers as HTLs in Organic Solar Cells

Since the breakthrough of organic solar cells in late 1958, their attention has been rapidly growing in the solar cell community owing to their flexibility, ease of processability, light weight, abundancy of raw materials, ease of synthesis of organic materials, and low fabrication or production costs [165,166]. Much of the success is due to the development of the bulk heterojunction (BHJ) solar cells which provides an enhanced charge separation path-length due to the increased donor-acceptor (D-A) interface, where the charges are separated efficiently [167]. As opposed to the organic-silicon hybrid heterojunction solar cells discussed in Section 4.2, where silicon acts as the main light absorber, OSCs utilize a blend mixture of conjugated donor and acceptor polymers (D-A) as the active layer responsible for absorbing light to generate excitons. The active layer is sandwiched in the middle of two electrodes having different work functions, ITO and metal as the front and back contact, respectively, as illustrated in Figure 3 under Section 4.1. Recently, due to the novel design of new organic donor and acceptor materials, innovative-engineered architectures, and device optimizations, single-junction OSCs have significantly improved their performances with PCEs exceeding 17% [168,169,170,171] and reaching 18.22%, to date [172]. As much as most of the research is centered around the design of novel donor and acceptor materials, the tremendous improvements of late are also owing to the utilities of suitable HTLs which form a significant constituent of OSCs and play an important role is enhancing both the PCE and stability of OSCs. Much as in organic-silicon hybrid heterojunction solar cells, the presence of the HTLs impede the head-on attachment between the active layer and the metal anode (ITO), thereby reducing carrier recombination at the front due to the electron blocking characteristics of the HTLs [135,136]. In addition, HTLs are capable of passivating surface defects and pinholes by regulating or modifying the surface of ITO, thereby decreasing the leakage of current, and thus increasing the *J_sc_* of the solar cell, which ultimately improves the PCE [173]. Amongst many, just as in organic-silicon hybrid heterojunctions solar cells, PEDOT:PSS is the most commonly and widely used solution-processable HTL in single-junction organic solar cells. This is because PEDOT:PSS is highly conductive, has a good optical transparency in the visible-to-near infrared which minimizes optical losses, can be easily dispersed in water, easily forms homogenous films using different deposition techniques, and has a work function of 5.2 eV close to ITO, which allows for good transport of holes between PEDOT:PSS and ITO [174,175,176,177,178]. Yuan et al. [179] fabricated the device with the structure ITO/PEDOT:PSS/PM6:Y6/PDINO/Al using PEDOT:PSS as the HTL and Y6 as the non-fullerene electron acceptor and achieved an efficiency of 15.7%. Anagnostou et al. [180] achieved an efficiency of 7.32% using PEDOT:PSS as HTL and Ca for transporting electrons. Moreover, Guo et al. [181] achieved an efficiency of 6.6% with the device architecture of ITO/PEDOT:PSS/P3HT:ICBA/Ca/Al, fabricated from non-halogenated solvents. Despite the merits exhibited by PEDOT:PSS and its extensive usage in conventional OSCs, it somehow also exhibits drawbacks that are a limiting factor in the performances and industrial uses of OSCS. The first factor is the hygroscopic character, as it can absorb moisture and oxygen which affects the long-term stability of the devices by promoting fast degradation at the ITO/PEDOT:PSS interface [182,183]. Secondly, the acidic nature of PEDOT:PSS tends to corrode the ITO surface which results in rough surface morphology, thereby inhibiting the free-flow and collection of holes and hammer over the device’s lifetime [184,185]. For example, Wu et al. [186] investigated the effect of trifluoroacetic acid (TFA) treatment of PEDOT:PSS layers on the performance and stability of organic solar cells. The results revealed that the device with pristine PEDOT:PSS as HTL remains with 1% of the initial efficiency after 63 days of storage, whereas the device treated with TFA retains 25% of its initial efficiency. In another example, Xu et al. [187] observed a decay of 72% of PCE after just 315 h for pristine device with PEDOT:PSS as HTL, whereas a 60% decay was observed for a device with solvent additives on PEDOT:PPS/ITO front contact after 315 h.

Due to these constraints experienced by devices fabricated from PEDOT:PSS as HTL, research has led to the design and development of alternative conjugated polymers to be used as HTL in substitution of PEDOT:PSS. Conjugated polyelectrolytes, which are polymers, composed of conjugated backbones and ionic pendant functionalities, have emerged as favorites and front runners to replace PEDOT:PSS. Similar to most conjugated polymers, the conjugated backbone of polyelectrolytes determines the optical and electrical behavior of the material, whereas the side chain ionic functionalities determine the solubility and the ability of forming homogenous films. Therefore, this implies that the electrical and optical properties of the polyelectrolytes can be tuned through structural modifications [188,189]. Moreover, polyelectrolytes meet the standard requirements for good HTLs such as high HOMO level for transporting holes and low LUMO level for blocking electrons, high transmittance in the visible region, and good solubility in friendly common laboratory solvents. The good solubility property allows for simplicity in depositing layers via solution processing for optoelectronic devices [190]. In contrast to PEDOT:PSS, polyelectrolytes are neutral and do not corrode the surface of the ITO, thereby increasing the lifetime or stability of the devices.

**Scheme 17:**
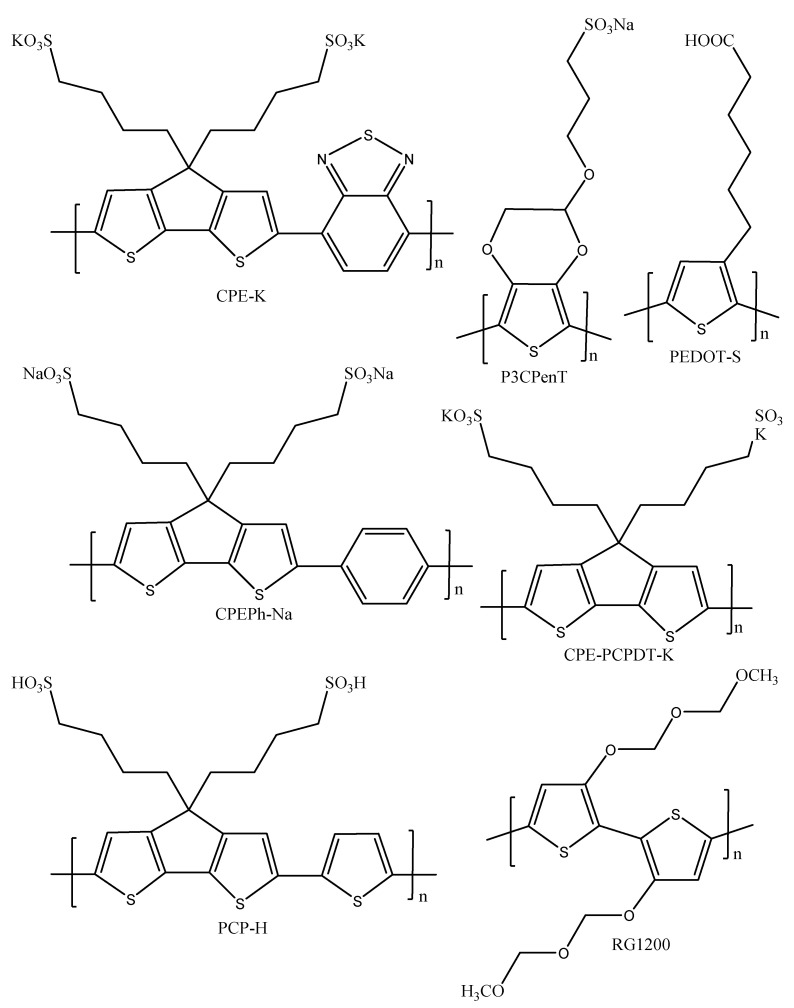
Structures of common conjugated polymer hole transporting layers in OSCs.

In addition, the presence of the pendant ionic functionalities permits the generation of surface dipoles permanently, thereby changing the work function of electrodes through the creation of interfacial dipoles at the electrode/active layer interface [191,192]. Due to these advantageous properties exhibited by polyelectrolytes, they have gained attention and are regarded as promising HTLs for extensive applications in OSCs. Moon et al. [193] fabricated a series of conventional devices based on CPE–K, CPE–Na, CPE–PCPDT–K polyelectrolytes as HTLs and compared them with the traditional device made from PEDOT:PSS. Polyelectrolytes-based devices achieved higher efficiencies compared to the conventional PEDOT:PSS-based organic device, with CPE–PCPDT–K producing the highest efficiency of 3.11%. The better performances from CPE-based devices were attributed to better charge transfer across the CPEs HTL/active layer interface, low surface roughness, and higher transmittance in the visible region. The common conjugated chemical structures of HTLs and their corresponding summary of device performances are illustrated in Scheme 17 and Table 5, respectively. In another study, Choi et al. [194] fabricated a series of conventional devices based on PFF, PFT, PFTSe polyelectrolytes as HTLs and compared them with the traditional device made from PEDOT:PSS. Polyelectrolytes-based devices achieved higher efficiencies compared to the conventional PEDOT:PSS-based organic device, with PFTSe producing the highest efficiency of 7.2%. The PFTSe device also demonstrated better air stability and long lifetime with about 70% retention of its initial PCE after air exposure of 480 h, whereas the PEDOT:PSS-based device lost 50% of its initial power in the same time frame. The improved long-term stability was ascribed to the neutral nature of the polyelectrolytes with a pH of 6.7–7.0 which does not corrode the ITO surface.

## 5. Conclusions

π-Conjugated polymers have attracted considerable and significant recognition in the area of solar cells owing to their flexibity, ease of synthesis, light weight, tunability of optical and electrical properties through structural modifications, and solution processability. At present, the challenge in solar cells is centered on finding the device with the highest PCE, long-term stability, and that comes at a lower production or fabrication cost in order to gain broad stream commercialization and large-scale industrial production. For these reasons, conjugated polymers have found extensive application in organic-silicon hybrid heterojunction solar cells and pure organic solar cells, to afford flexible devices at a lower cost. Tremendous progress has been made in optimizing device structures, designing and developing new conjugated polymers with desirable properties, and engineering front contact interfaces. This review focused particularly on the significant progress made in the employment of conjugated polymers as HTLs in organic-silicon hybrid heterojunction solar cells and organic solar cells, and how they affect the PCE and stability of the devices. In organic-silicon hybrid heterojunction solar cells, the PCE of the devices using HTLs such as PEDOT:PSS and P3HT in place of (*p*) a-Si:H has dramatically increased over the years. In order to achieve high efficiencies, the conjugated polymers have to have high transmittance in the visible to near-infrared region, high conductivity, and high hole mobility. Despite the impressive progress made, these cells suffer from long-term stability owing to the hygroscopic character of PEDOT:PSS and photo-oxidation at the anode interface that results in total degradation of the polymeric p-type material. One strategy employed to overcome this challenge is the use of the p-type conjugated polymer in the rear side of the device in an inverted configuration, widely referred to as the backPEDOT concept, in order to avoid parasitic absorption. Using this backPEDOT strategy, organic-silicon hybrid solar cells have shown excellent PCEs close to PCEs achieved by traditional silicon heterojunction solar cells and improved solar cells lifetimes. On the other hand, in OSCs, different conjugated polymers have been successfully used as HTLs. PEDOT:PSS and polyelectrolytes are the top favorites. Devices fabricated from PEDOT:PSS HTL suffer from long-term stability due to the acidic character of PEDOT:PSS which corrodes the ITO anode, thereby reducing its lifetime. In contrast, polyelectrolytes-based devices are robust, stable, and produce reasonably high efficiencies. This is because they are neutral and do not corrode the anode ITO, thereby creating a smooth morphological surface for the transfer and collection of holes by the ITO. Generally, although great progress has been seen in the incorporation of conjugated polymers in the various solar cell types, there is still a long way for cells incorporating polymeric materials to realize commercialization and large-scale industrial production due to the shortcomings in the stability of the polymers and their low conductivities compared to traditional silicon-based solar cells. Although these challenges exist, the current fast-growing significant progress, together with future innovative designs for new conjugated polymeric materials and solar cell architectures, provide enough room for future promise and hope that commercialization will soon emerge.

## Figures and Tables

**Table 1:** Examples of common p-type dopants of conjugated polymers.

Dopant Name	Conjugated Polymer-Dopant System	Conductivity (S.cm^−1^)	Dopant Counter Ion	Chemical Nature	Ref.
Ferric (III) Chloride	P3HT-FeCl_3_	6.3 × 10^1^	Cl^−^	Inorganic	[44]
Tartaric acid (TA)	Pani-TA	0.2	C_4_H_5_O_6_^−^	Organic	[45]
Chloroauric acid	P3HT-HAuCl_4_	71.7	AuCl_4_^−^	Inorganic	[46]
Oxalic acid (OA)	Pani-OA	0.5	C_2_HO_4_^−^	Organic	[47]
Perchloric acid	Pani-HClO_4_	4.68 & 2.72	ClO_4_^−^	Inorganic	[48]
p-Hydroxybenzene sulfonic acid (HBSA)	PPY-HBSA	11.0	HAS^−^	Organic	[49]
Arsenic pentafluoride	PPV-AsF_5_	1.0	AsF_5_^−^	Inorganic	[50]
Poly(2-methyoxyaniline-5-sulfonate) (PMSH)	PPY-PMSH	2.0	PMS^−^	Polymeric	[51]
p-Dodecylbenzene sulfonic acid (DBSA)	PPY-DBSA	2.0	DBS^−^	Organic	[49]
Poly(styrene sulfonic acid) (PSSA)	PEDOT-PSSA	0.1–10.0	PSS^−^	Polymeric	[52]
p-Toluene sulfonic acid (TSA)	Pani-TSA	1.2 × 10^−2^	Tosylate	Organic	[53]
β-Naphthalene sulfonic acid (NSA)	PPY-NSA	18.0	NS^-^	Organic	[49]

**Table 2:** List of few common n-type dopants of conjugated polymers.

Dopant Name	Conjugated Polymer-Dopant System	Conductivity (S.cm^−1^)	Dopant Counter Ion	Chemical Nature	Ref.
Sodium	PA-Na	10^4^	Na^+^	Inorganic	[54]
Potassium	Polyphenylene-K	10^3^	K^+^	Inorganic	[54]
Lithium	Polyphenylene-Li	10^3^	Li^+^	Inorganic	[54]
Lithium naphthanilide	PA-Li naphthanilide	200	Li^+^	Inorganic	[55]
Sodium naphthanilide	PA-Na naphthanilide	25	Na^+^	Inorganic	[55]

**Table 3:** Examples of conjugated polymers produced by oxidative polymerization.

Monomer	Conjugated Polymer System	Polymerization Reaction System/Content	Oxidative Polymerization Type	Application	Ref.
3-hexylthiophene (3HT)	2,3,4,5-tetrathienylthiophene-*co*-poly(3-hexylthiophene-2,5-diyl) (TTT-*co*-P3HT)	Palladium –catalysed synthesis of TTT, followed by polymerization of 3HT using FeCl_3_ in CHCl_3_	Chemical	OSC	[59]
3,4-ethylenedioxythiophene (EDOT)	G1Poly(propylene thiophenoimine)-co-poly(3,4-ethylenedioxythiophene) Star copolymer (G1PPT-*co*-PEDOT)	Schiff base condensation of G1PPT, followed by polymerization using (NH_4_)_2_S_2_O_8_ in H_2_O and CHCl_3_	Chemical	Potential application on OSC	[18]
Pyrrole (Py)	G1Poly(propylene imine)-*co*-Polypyrrole star copolymer (G1PPI-*co*-PPY)	Schiff base condensation of G1PPI-2Py, followed by polymerization using (NH_4_)_2_S_2_O_8_ in H_2_O and CHCl_3_	Chemical	Potential application in sensors, and membranes	[60]
3-hexylthiophene (3HT)	Poly(3-hexylthiophene)-graft-polypropylene (P3HT-g-PP)	Grafting of PS on the surface of PP, followed by polymerization using FeCl_3_ in hexane	Chemical	Not mentioned	[61]
3-hexylthiophene (3HT)	Poly(3-hexylthiophene)-graft-polypropylene (P3HT-g-PP)	Grafting of PEPT on the surface of PP, followed by polymerization using FeCl_3_ in hexane	Chemical	Not mentioned	[62]
3,4-ethylenedioxythiophene (EDOT)	Polypropylene-graft-poly(acrylic acid)/poly(3,4-ethylenedioxythiophene) (PP-g-PAA/PEDOT)	Photo- induced graft polymerization of AA from PP fiber surfaces, followed by polymerization using FeCl_3_ in ethanol	Chemical	Gas sensing	[63]
3-hexylthiophene (3HT)	Poly(3-hexylthiophene) (P3HT)	Ultrasound treatment of FeCl_3_/hexane mixture, followed by introduction of 3HT and magnetic stirring	Chemical	Not mentioned	[64]
3,4-ethylenedioxythiophene-methanol (EDOT-HPG)	Hyperbranched polyglycerol-grafted-poly(3,4-ethylenedioxythiophene) (PEDOT-g-HPG)	Anionic ring opening reaction on glycidol on the PEDOT-MeoH, followed by deposition of EDOT-HPG on a bare GCE electrode from an aqueous solution containing 100 mM LiClO_4_ and 60 mM EDOT-HPG	Electrochemical	Protein detection in complex biological media	[68]
3,3-bis(cyclohexylmethyl)-3,4-dihydro-2H-thieno [3,4-b][1,4] dioxepin (ProDOT-CycHex_2_)	Poly(3,3-Bis(cyclohexylmethyl)-3,4-Dihydro-2H-Thieno [3,4-b][1,4] Dioxepin) (P_22_ProDOT-CycHex_2_)	electrolytic solution of 0.1 M tetrabutylammonium hexaflourophosphate (TBAH) dissolved in a mixture of acetonitrile (ACN) and dichloromethane (DCM)	Electrochemical	Candidate for electrochromic devices	[69]
5-(2-ethylhexyl)-1,3-di(thiophen-2-yl)-4H-thieno [3,4-c] pyrrole-4,6(5H)-dione (TTPT)	Poly(5-(2-ethylhexyl)-1,3-di(thiophen-2-yl)-4H-thieno [3,4-c] pyrrole-4,6(5H)-dione) (TTPT)	surface of a platinum electrode in an electrolytic solution of 0.1 M TBAH in a solvent mixture of ACN and DCM	Electrochemical	Candidate for Optoelectronic devices	[70]
Pyrrole (Py)	G2poly(propylene imine)-*co*-polypyrrole star copolymer (G2PPI-*co*-PPY)	Surface of a platium electrode in electrolytic aqueous solution n of 0.1 M LiClO_4_ containing pyrrole monomer and pyrrole-functionalized dendrimer	Electrochemical	Not mentioned	[71]
3,4-ethylenedioxythiophene (EDOT)	G1Poly(propylene thiophenoimine)-*co*-polythiophene (G1PPT-*co*-PEDOT)	Surface of gold (Au) electrode in an aqueous electrolytic solution consisting 0.1 M LiClO_4_ and sodium dodecyl sulphate	Electrochemical	aptamer biosensor	[72]
3-hexylthiophene (3HT)	G3Poly(propylene thiophenoimine)-*co*-poly(3-hexylthiophene)	Surface of Au electrode in an electrolytic solution of 0.1 M Bu_4_NClO_4_ dissolved in ACN containing 3HT monomer	Electrochemical	Phenanthrene sensor	[73]

**Table 4:** Research progress of organic-silicon hybrid heterojunction solar cells using conjugated polymer as HTLs.

Solar Cell Hybrid Heterojunction Architecture	*J_sc_* (mA/cm^2^)	*V_oc_* (mV)	*FF* (%)	PCE (%)	Ref.
ITO/PEDOT:PSS-Go/SiNWs/(*n*) c-Si/Ag/Ti	31.00	518	59.6	9.57	[140]
Ag/PEDOT:PSS-DMSO/(*i*) a-Si:H/(*n*) c-Si/(*i*) a-Si:H/(n^+^) a-Si:H/a-SiC:H/Ti/Al	30.97	600	59.4	11.04	[141]
ITO/PEDOT:PSS/SiNHs/(*n*) c-Si/n^+^ layer/Ti/Ag	36.81	492	66.3	12	[142]
Ag-grid/PEDOT:PSS/(*n*) c-Si/(i) a-Si:H/(*n*) a-Si:H/Al	36.5	634	70	16.2	[143]
Ag/PEDOT:PSS-Gr/(*n*) c-Si/Al	26.64	530	64	9.05	[145]
ZnS/Ag/MoO_3_/PEDOT:PSS/*n*-SiNCs/Al	24.21	400	53	5.12	[146]
Cu-grid/P3HT/(*n*) SiNPs/In/Ga	37.6	457	54	9.2	[147]
Ti/Al-grids/Au/MoO_3_/P3HT/CH_3_-Monolayer/poly-Si/SiO_2_+SiN_3_N_4_+SiO_2_/Glass	24.3	552	49.8	6.6	[153]
Al/SiNx/Al_2_O_3_/n^+^-FSF/(*n*) c-Si/SiOx/PEDOT:PSS/Ag	39.7	653	67.2	17.4	[154]
ITO/(*n*) a-Si:H/(*i*) a-Si:H/(*p*) c-Si/PEDOT:PSS/Ag	32	688	74.3	16.2	[155]
Ag-grid/PEDOT:PSS/SiO_2_/(*n*) c-Si/(*i*) a-Si:H/(n^+^) a-Si:H/ITO/Ti/Ag	31.9	663	70	14.8	[158]
Ag-grid/PEDOT:PSS-5wt% DMSO/*n*-SiNWs/Al	30.1	569	55	9.3	[159]
Ag-grid/PEDOT:PSS-6wt% ethylene glycol/*n*-Si/rubrene:DMSO/Ag	28	609	69	11.9	[160]
Ag-grid/PEDOT:PSS/Dopamine@graphene/*n*-Si/PC61BM/Al	32.64	623	64.6	13.15	[161]
Semi-transparent Au/P30T/SiOx/(*n*) c-Si/(*i*) a-Si:H/(n^+^) a-Si:H/metal	-	500	-	9.6	[135]
Ag-grid/PEDOT:PSS-5wt% DMSO-0.1wt% Tritocn X 100/*n*-SiNWs/Al	27.32	598	75.7	12.32	[162]
Glass/SiNx/(*n*) mc-Si/Al_2_O_3_/PEDOT:PSS/Ag	25.4	604	67.4	10.3	[163]
Ag-grid/PEDOT:PSS-7% EG/*n*-Si/Ag	15.7	598	58	5.1	[164]

**Table 5:** Organic solar cells research work focused on conjugated polymers as HTLs.

Organic Solar Cell Architecture	*J_sc_* (mA/cm^2^)	*V_oc_* (mV)	*FF* (%)	PCE (%)	Ref.
ITO/PEDOT:PSS/PM6:Y6:PDINO/Al	25.3	830	74.8	15.7	[179]
ITO/PEDOT:PSS/PTB7:PC_71_BM/Ca/Ag	16.32	760	59.6	7.32	[180]
ITO/PEDOT:PSS/P3HT:ICBA/Ca/Al	10.3	850	75	6.6	[181]
ITO/CPE-PCPDT-K/P3HT:PCBM/Al	7.88	610	63	3.11	[193]
ITO/PFTSe/PTB7:PC_71_BM/PFN/Al	14.4	677	69	7.2	[194]
ITO/PCPDffPhSO_3_K/PTB7-Th:PC_71_BM/Al	17.88	782	65.7	9.4	[195]
ITO/PCP-H/J52-2F:IT-M/PFN-Br/Al	18.4	960	73	12.8	[196]
ITO/PFS/PBDT-T:IT-M/PFN-Br/Al	17.2	921	70	11	[197]
ITO/PCP-Na/PBDT-TSI:PC_71_BM/Mg/Al	17.46	803	70.6	9.89	[198]
ITO/PCP-3B/PBDT-T:IT-M/NDI-n/Al	15.5	910	68	9.67	[199]
ITO/PEDOT:PSS/PTB7-Th:PC_71_BM/ZnO/CPEPh-Na/PTB7-Th:PC_71_BM/AL	11.1	1540	66	11.3	[200]
ITO/PFS/PBDT-TSI:PC_71_BM/Mg/Al	18.2	794	66.6	9.60	[201]
ITO/RG1200/P3HT:PCBM/Ca/Al	8.1	580	60.3	2.82	[202]
ITO/p-PFP-HD/PTB7-Th:PC_71_BM/PFN/Al	16.3	780	77	9.03	[203]
ITO/p-PFP-O/PTB7-Th:PC_71_BM/PFN/Al	16.6	780	70	9.2	[204]
ITO/PEDOT-S/P3TI:PC_71_BM/LiF/Al	12.89	690	58	5.12	[205]

## Data Availability

The data presented in this study are available on request from the corresponding author.
